# An objective index for spinal cord compression on computed tomography in Thoroughbred horses

**DOI:** 10.1002/vms3.767

**Published:** 2022-02-13

**Authors:** Taro Kondo, Fumio Sato, Nao Tsuzuki, Chun‐Jen Chen, Kazutaka Yamada

**Affiliations:** ^1^ School of Veterinary Medicine Azabu University Sagamihara Japan; ^2^ Equine Research Institute Japan Racing Association Shimotsuke Japan; ^3^ Department of Veterinary Medicine Obihiro University of Agriculture and Veterinary Medicine Obihiro Japan

**Keywords:** an objective index, cervical vertebral malformation, computed tomographic myelopathy, Thoroughbred

## Abstract

**Background:**

Computed tomographic myelography can be a useful tool for evaluating vertebral canal stenosis. However, an index of spinal cord compression is yet to be established.

**Objectives:**

This observational descriptive study aimed to establish an index for spinal cord compression using computed tomography (CT).

**Methods:**

Twenty‐three Thoroughbred horses (age, 155–717 days; weight, 205–523 kg) with suspected cervical vertebral malformation were subjected to computed tomographic myelography in dorsal recumbency using large‐bore gantry CT to define the entire cervical vertebrae from C1 to C7. Subsequently, the height of the spinal cord was measured in the sagittal plane reformatted using curved multi‐planar reformation (MPR), thereby comparing it with stenotic ratio (i.e. dividing the area of spinal cord by that of the subarachnoid space) measured in the transverse plane. The measurement was performed at the level of each of six intervertebral spaces, for a total of 138 sites. Accordingly, the appropriate cut‐off value for spinal cord height was determined using the receiver‐operating characteristic curve, from which the area under the curve with 95% confidence interval was estimated.

**Results:**

The spinal cord compression cut‐off value was 7.06 mm, with an area under curve of 0.84. A weak relationship was observed between spinal cord height and stenotic ratio (*R* ^2^*^^* = 0.08, *p* < 0.05).

**Conclusions:**

Following curved MPR, a cut‐off value of 7.06 mm may serve as an index for spinal cord compression.

## Graphical abstract

Measurement of spinal cord height can provide objective index for cervical vertebral malformation.

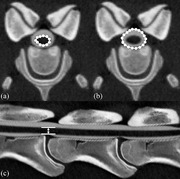

## 1 INTRODUCTION

Developmental orthopaedic diseases, including angular limb deformities, osteochondrosis dissecans, subchondral bone cysts and cervical vertebral malformation (CVM), often affect young adult Thoroughbred horses. Although riding horses with CVM have occasionally received surgical treatment (Fürst et al., 2019), racehorses seldom undergo treatment because they are not expected to recover their full racing potential even with complete surgical treatment. Therefore, owners with racehorses are affected by severe myelopathy incur economic losses (Hoffman & Clark 2013). Early diagnosis and prognostic determination are some countermeasures for reducing such economic loss.

X‐ray examination, the first and relatively easy‐to‐use diagnostic tool for orthopaedic disorders, is widely available at many veterinary clinics. Findings on cervical vertebral plain radiographic projections, including subluxation and degenerative joint disease, are suggestive of abnormalities (van Biervliet et al., 2006; Levive et al., 2007). The use of the intervertebral ratio was a limitation of radiographic assessment as it was reported to be unreliable, with variations of 5%–10% (Scrivani et al., 2011). In addition, radiography is unable to identify spinal cord compression given the similar X‐ray attenuation of the spinal cord and cerebrospinal fluid. Therefore, myelography, wherein a contrast agent is injected into the subarachnoid space, has been utilised to determine spinal cord compression (Aleman et al., 2014; Rose et al., 2007; van Biervliet et al., 2006). Although lateral cervical myelogram can reveal the site and degree of dorsal and ventral compressions, determining lateral canal stenosis remains difficult (van Biervliet et al., 2006; Szklarz et al., 2019). Moreover, the interpretation of a myelogram is somewhat subjective (van Biervliet et al., 2004).

Magnetic resonance imaging (MRI) is a useful tool for spinal cord imaging that does not require injection of contrast agent into subarachnoid space. Thus, MRI plays an important role in the diagnosis of spinal cord disorders among small animals (Wisner & Zwingenberger 2015). However, MRI of the equine neck has been described only in post‐mortem specimens (Janes et al., 2014, 2015; Mitchell et al., 2012; Sleutjens et al., 2014;). This is because it requires a gantry size that is as wide as the shoulder of horses. Nevertheless, the gantry size for MRI was originally designed to accommodate a human‐sized trunk, and even foals have a shoulder size much larger than a human trunk, suggesting that the entire neck cannot fit into the gantry. For this reason, the use of MRI for the entire C1 to C7 spinal cord in living horses has not been reported.

Equine computed tomography (CT) was first introduced in 1987 (Barbee et al., 1987). However, it was not until 2010s that the use of CT examination of the spinal cord began in living horses (Veraa et al., 2016). Contrast agent injection into the subarachnoid space was necessary to delineate the dura mater and pia mater. Thus far, CT myelography has provided useful information on live horses affected with CVM (Lindgren et al., 2020). One study had quantitatively evaluated stenosis using CT myelography by calculating the stenotic ratio (i.e. dividing the area of spinal cord by that of subarachnoid space). The stenotic ratio is used to evaluate the area of the spinal cord relative to the area of the subarachnoid space, with larger values indicating lesser subarachnoid space relative to the spinal cord size (Yamada et al., 2016). However, in this previous study, calculation of the stenotic ratio utilised images with a symmetric appearance of the transverse plane using a multi‐planar reformation (MPR) procedure by adjusting the sagittal and dorsal planes simultaneously. Therefore, delineating the area of spinal cord and of subarachnoid space is laborious.

**FIGURE 1:**
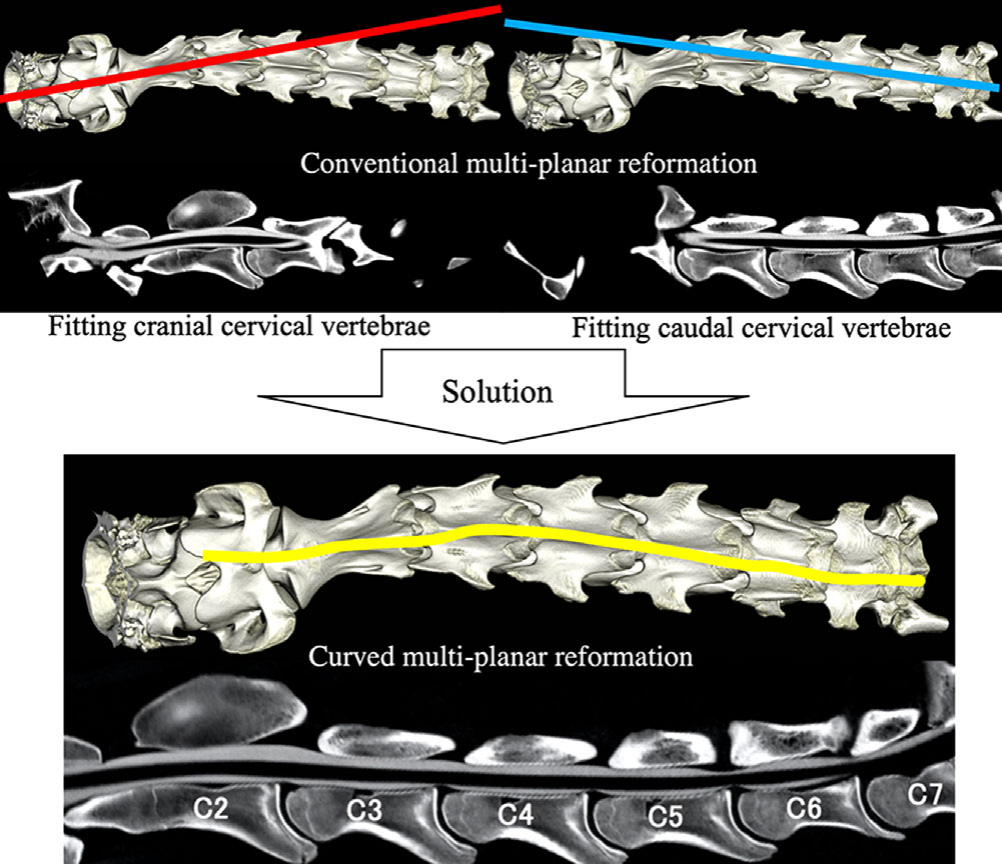
Conventional method was impossible to use as the angled neck configuration fit C1 to C7 into one single plane. Curved multi‐planar reformation reformatted the image of the angled neck along the structure in one single plane

CT images can be reformatted from the transverse plane to the dorsal and sagittal planes in one acquisition. Simultaneous comparison in each vertebra is difficult given that the transverse CT plane is obtained differently for each cervical vertebral level. However, the true sagittal plane can simultaneously compare each intervertebral site, but during CT image acquisition in horses, the angled neck configuration could not fit C1 to C7 into one plane. Curved MPR reformats the image of the angled neck along the structure in one single plane (Figure 1).

**FIGURE 2:**
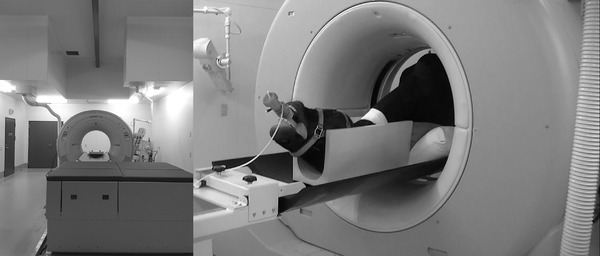
An equine computed tomographic scanner in which the gantry is self‐propelled with the opening gantry size of 90 cm. This custom‐made patient table was made of radiolucent carbon fibre, which did not move during the scan. The crane was set on the ceiling to lift the horses at Obihiro University of Agriculture and Veterinary Medicine

According to the literature, equine CT is performed in lateral recumbency (Gough et al., 2020; Lindgren et al., 2020). However, lateral recumbency often results in an angled neck due to the difference between the heights of the skull and shoulder on the CT table (Gough et al., 2020; Lindgren et al., 2020). This study used an equine CT scanner in which the gantry had an opening gantry size of 90 cm. This custom‐made equine CT patient table was made of radiolucent carbon fibre, which can withstand a load of 500 kg (Figure 2). Thus, we positioned the horse on the CT patient table, with the patient in dorsal recumbency with the neck straighter than that in lateral recumbency. In addition, curved MPR was used for image processing. This observational descriptive study aimed to establish an objective index for spinal cord compression.

## 2 MATERIALS AND METHODS

### 2.1 Study design, sample and setting

This study was an observational descriptive study. Twenty‐three Thoroughbred horses (age, 155–717 days; weight, 205–523 kg) exhibiting ataxia with suspected CVM leading to poor athletic performance underwent CT myelography from June 2015 to October 2019 in Obihiro University of Agriculture and Veterinary Medicine. The horses’ weight and age were obtained from medical records. The study protocol was approved by the Animal Experiment and Welfare Committee of Obihiro University of Agriculture and Veterinary Medicine (No. 27–127).

### 2.2 Computed tomographic myelography

An indwelling IV catheter was placed into the jugular vein. The horses were premedicated intravenously with 5 μg/kg medetomidine hydrochloride. Then, anaesthesia was induced using intravenous administrations of 0.03 μg/kg diazepam and 4 mg/kg thiamylal. Afterwards, guaifenesin (25 mg/kg) was rapidly infused until the horse became ataxic and was subsequently maintained with a triple drip, which was a mixture of 1000 ml guaifenesin (100 mg/ml), 25 ml xylazine (20 mg/ml) and 100 ml ketamine (20 mg/ml), administered at a rate of 2 ml/kg/h. A 21‐gauge spinal needle was then placed into the subarachnoid space via the atlanto‐occipital junction. Cerebrospinal fluid was allowed to drain out of the subarachnoid space for 2 min, after which the same volume as drained cerebrospinal fluid of contrast agent (iohexol, 140 mgI/ml) (Teva Pharmaceutical Industries, Tokyo, Japan) was injected into the subarachnoid space. Subsequently, the horse's head was lifted for 5 min, after which the horse was placed in dorsal recumbency with the neck in a neutrally extended position on a custom‐made equine patient table for CT. The equine CT gantry moved the animal through the patient table, which does not move during the scan. CT myelograms were obtained using a 16‐row multidetector CT (Aquilion SP, Canon, Ohtawara, Japan), tube voltage of 135 kV, tube current of 300 mA, slice thickness of 0.5 mm, helical pitch of 0.93 and maximum scanning time of 46 s. Images were reconstructed using bone algorithm.

**FIGURE 3:**
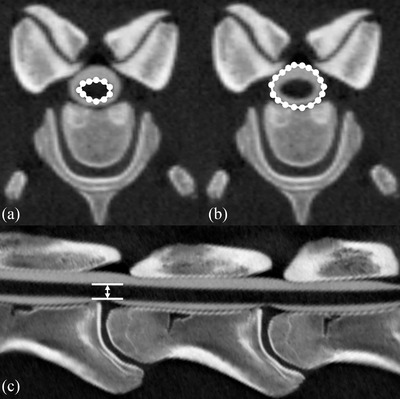
Measurement of the spinal cord area (a) and that subarachnoid space in the transverse plane (b). Measurement of length between dorsal aspect and vertebral aspect of spinal cord in the sagittal plane (c)

The stenotic ratio was calculated as the area of spinal cord divided by the area of the subarachnoid space that includes the area taken up by the spinal cord at the level of intervertebrae in the transverse plane (Yamada et al., 2016). The sagittal plane was fixed using curved MPR image‐processing software (Virtual Place; Canon). Subsequently, the spinal cord height was measured between the dorsal and the ventral aspects of the spinal cord at the level of each intervertebra in the sagittal plane using curved MPR (Figure 3). The spinal cord measurement was used in the image‐processing software (OsiriX‐N; Newton Graphics, Sapporo, Japan).

**FIGURE 4:**
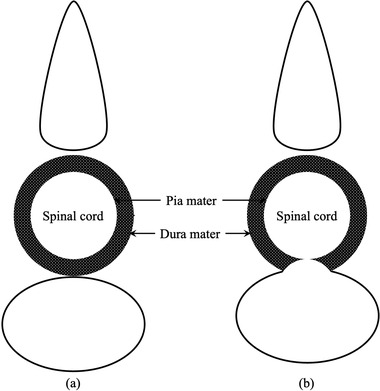
Impingement was defined as a deficit of contrast agent between the border of the dura mater and the border of the pia mater. (a) Without impingement (b) With impingement

The images were read by board certified equine veterinarians by the Japanese Society of Equine Science (K.Y, F.S). Moreover, board certified equine veterinarians drew the outlines of the subarachnoid space and spinal cord. Impingement was defined as a deficit of contrast agent between the border of the dura mater and the border of the pia mater (Figure 4) (Lindgren et al., 2020). The animals were then divided into two groups: those with and those without evidence of spinal cord impingement on the transverse planes.

### 2.3 Statistics

Data were statistically analysed using the JMP v.15.2.0 software (SAS Institute Japan, Tokyo, Japan). The appropriate cut‐off value for spinal cord height was determined using the receiver‐operating characteristic (ROC) curve, from which the area under the curve (AUC) with 95% confidence interval, in addition to the sensitivity, specificity and accuracy, were determined.

The relationship between spinal cord height in the sagittal plane and stenotic ratio in the transverse plane were determined. These measurements were performed both with and without spinal cord impingement. Spinal cord height and stenotic ratio measurements were compared by correlating statistical significance and *χ* ^2^ regression analysis (*p *< 0.05). Moreover, the relationship between spinal cord height without impingement and age in days were determined using correlation statistical significance and *χ* ^2^ regression analysis (*p* < 0.05). These data were analysed using Microsoft Excel for Mac 2019 (Microsoft Japan, Tokyo, Japan).

## 3 RESULTS

CT images of the entire cervical vertebral column from C1 to C7 were obtained in all 23 live horses. Contrast agent leakage from subarachnoid space to subdural space was observed in seven out of 23 cases. Spinal cord height in each case was measured in six intervertebral space sites, for a total of 138 sites. Myelograms were evaluated in 138 spinal cord intervertebral sites, of which 37 had spinal cord impingement.

**FIGURE 5:**
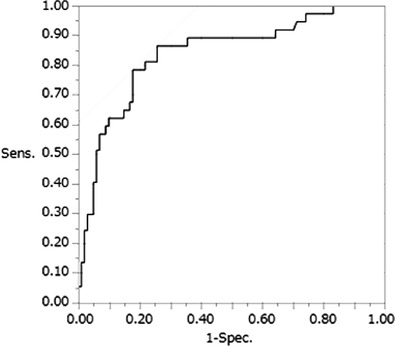
The cut‐off value for spinal cord height was determined at 7.06 mm, based on receiver‐operating characteristic curve analysis

No significant difference in spinal cord height among intervertebral space sites was observed. The cut‐off value for spinal cord height was determined at 7.06 mm based on ROC curve analysis, with an AUC = 0.84, sensitivity = 86.5% and specificity = 74.3%. Moreover, the accuracy of positive and negative prediction of spinal cord impingement was 77.5% (Figure 5).

**FIGURE 6:**
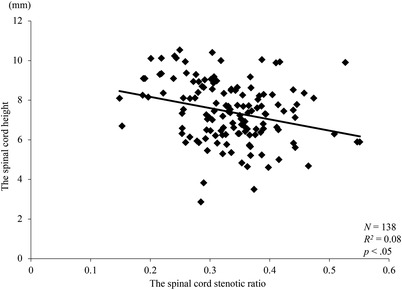
Relationship between spinal cord height in the sagittal plane and stenotic ratio of the spinal cord in the transverse plane

**FIGURE 7:**
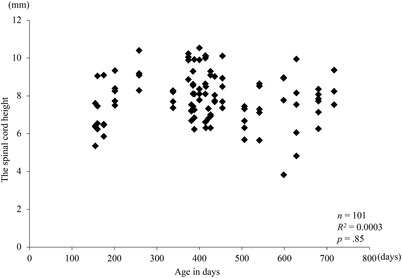
Relationship between spinal cord height and age in days. Squares indicate no spinal cord compression. Considering that the central nervous system of a 155‐day‐old horse has stopped, this index could be applied to horses between 155 and 717 days old

A weak negative correlation was found between spinal cord height in the sagittal plane and stenotic ratio in the transverse plane (*R* ^2^*^^* = 0.08, *p *< 0.05) (Figure 6). Moreover, no significant difference between spinal cord height and age in days was observed (*R* ^2^*^^* = 0.0003, *p *= 0.85) (Figure 7).

## 4 DISCUSSION

**FIGURE 8:**
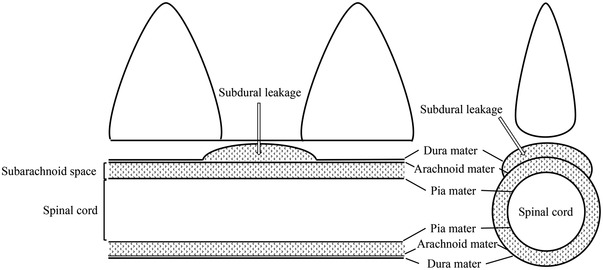
Subdural contrast agent leakage may have led to the underestimation of the stenotic ratio

In dorsal recumbency, using large‐bore gantry CT was able to define the entire cervical vertebrae from C1 to C7. The conventional sagittal CT plane cannot accommodate C1 to C7 in one plane due to angular deviations present. This was addressed using curved MPR given that it reformats the image of the angled neck along the structure in one single plane. Additionally, we were able to simultaneously compare each level of apparent spinal cord impingement. Producing a symmetric appearance of the transverse plane by adjusting sagittal and dorsal planes simultaneously was unnecessary. Furthermore, subdural contrast agent leakage may have led to the underestimation of the stenotic ratio (Figure 8), given that it led to considerably increased areas of subarachnoid space being measured, thereby resulting in a low stenotic ratio (Yamada et al., 2016). Spinal cord height measurements can be used to evaluate dorsoventral spinal cord compression even if there is subdural contrast agent leakage. Subdural contrast agent generally leaks to the lateral side extension. Therefore, contrast agent leakage does not affect spinal cord height measurement in the sagittal plane.

Considering that no diagnostic index has thus far been used for CT myelographic diagnosis of CVM, the cut‐off value of 7.06 mm obtained herein may be considered an index, with an AUC of 0.84. As such, a spinal cord height of <7.06 mm can suggest spinal cord compression. Among the 37 sites measured with spinal cord impingement, only 13% were inconsistent, with a cut‐off value of 7.06 mm in the curved MPR sagittal plane. As such, the measurement's diagnostic accuracy was 0.84, calculated using the AUC, considering that spinal cord impingement could be detected in 32 sites, missing only in five sites.

Curved MPR allows the visualisation of entire cervical vertebral structures with minimal modification of the original data (Kanitsar et al., 2002). Curved MPR sagittal planes have thus far been used only for observing the location of spinal cord compression in veterinary medicine (Yamada et al., 2016). This image‐processing method has been well established and is used for measuring various vessels in humans (Nam et al., 2017). Hence, it was considered that the measurement obtained using a curved MPR image was accurate.

Although dorsolateral compression of the spinal cord can be barely observed in the sagittal plane, we observed a weak but significant relationship between spinal cord height in the sagittal plane and stenotic ratio measured in the transverse plane. Thus, spinal cord height can be used for evaluating spinal cord compression. Measuring the area of spinal cord and subarachnoid space is laborious. However, measuring the height of the spinal cord is relatively simple. Therefore, evaluation of the spinal cord used in this study can be more practical. No significant association between spinal cord height and age in days was observed among 155 to 717‐day‐old horses. Spinal cord of a 155‐day‐old horse has already stopped rapid growth; hence, this index could be applied to horses between 155 and 717 days old.

A limitation of this study is that large‐bore gantry CT was inevitably applied to equine CT. This study used a large‐bore human bariatric CT scanner with a heat capacity of 7.5 MHU. Given the large X‐ray tube heat capacity of this CT unit, the entire spinal cord, maximum 735 mm, could be scanned in one acquisition.

The horses were placed in dorsal recumbency on a custom‐made radiolucent patient table. We consider that dorsal recumbency allows the neck to be placed straight on the CT patient table compared with lateral recumbency. This is the first report in which the horse was placed in dorsal recumbency with the neck during CT. The second limitation, the dorsal recumbency was that the horses were placed with the neck in a neutrally extended position on an equine patient table for CT. Therefore, cervical spinal cord compression when the horse neck was placed in the flexion position was not evaluated. It is open to discussion that the flexion position promotes the detection of cervical spinal cord compression in the mid‐cervical region but also substantially increases the frequency of false‐positive diagnoses (van Biervliet et al., 2004; Lindgren et al., 2020). Further assessment of spinal cord compression in the flexion position is warranted in the future.

## 5 CONCLUSIONS

Following curved MPR, a cut‐off value of 7.06 mm may serve as an index for spinal cord compression.

## CONFLICT OF INTEREST

The authors declare no conflict of interest.

## ETHICS STATEMENT

This study protocol was approved by the Animal Experiment and Welfare Committee of Obihiro University of Agriculture and Veterinary Medicine (No. 27–127).

## AUTHOR CONTRIBUTIONS

*Conceptualisation, data curation, formal analysis, investigation, methodology, visualisation, writing—original draft preparation and writing—review and editing*: Taro Kondo. *Data Curation, investigation, resources and writing—review and editing: fumio sato. data curation, investigation, resources and writing—review and editing*: Nao Tsuzuki. *Data curation, formal analysis, resources and writing—review and editing*: Chun‐Jen Chen. *Data curation, funding acquisition, investigation, project administration, resources, supervision and writing—review and editing*: Kazutaka Yamada.

### PEER REVIEW

The peer review history for this article is available at https://publons.com/publon/10.1002/vms3.767.

## Data Availability

The data used to support the findings of this study are available from the corresponding author upon request.
